# How do person‐centered outcome measures enable shared decision‐making for people with dementia and family carers?—A systematic review

**DOI:** 10.1002/trc2.12304

**Published:** 2022-06-06

**Authors:** Jesutofunmi Aworinde, Clare Ellis‐Smith, Juliet Gillam, Moïse Roche, Lucy Coombes, Emel Yorganci, Catherine J. Evans

**Affiliations:** ^1^ Cicely Saunders Institute of Palliative Care, Policy & Rehabilitation King's College London London UK; ^2^ Division of Psychiatry University College London London UK; ^3^ The Royal Marsden NHS Foundation Trust Sutton UK; ^4^ Sussex Community NHS Foundation Trust Brighton UK

**Keywords:** communication, decision‐making, dementia, outcome assessment, patient‐centered care, quality of life

## Abstract

**Objectives:**

To identify published evidence on person‐centered outcome measures (PCOMs) used in dementia care and to explore how PCOMs facilitate shared decision‐making and improve outcomes of care. To build a logic model based on the findings, depicting linkages with PCOM impact mechanisms and care outcomes.

**Design:**

Mixed‐methods systematic review. We searched PsycINFO, MEDLINE, CINAHL, and ASSIA from databases and included studies reporting experiences and/or impact of PCOM use among people with dementia, family carers, and/or practitioners. Groen Van de Ven's model of collaborative deliberation informed the elements of shared decision‐making in dementia care in the abstraction, analysis, and interpretation of data. Data were narratively synthesized to develop the logic model.

**Setting:**

Studies were conducted in long‐term care, mixed settings, emergency department, general primary care, and geriatric clinics.

**Participants:**

A total of 1064 participants were included in the review.

**Results:**

Ten studies were included. PCOMs can facilitate shared decision‐making through “knowing the person,” “identifying problems, priorities for care and treatment and goal setting,” “evaluating decisions”, and “implementation considerations for PCOM use.” Weak evidence on the impact of PCOMs to improve communication between individuals and practitioners, physical function, and activities of daily living.

**Conclusions:**

PCOMs can enable shared decision‐making and impact outcomes through facilitating collaborative working between the person's network of family and practitioners to identify and manage symptoms and concerns. The constructed logic model demonstrates the key mechanisms to discuss priorities for care and treatment, and to evaluate decisions and outcomes. A future area of research is training for family carers to use PCOMs with practitioners.

## 1 INTRODUCTION

Dementia is a leading global cause of serious health‐related suffering, with an estimated increase of 246% over the next 40 years.^1^ As the number of people living with dementia is rising so is the number of people dying with it, with deaths estimated to increase from 59,000 to 219,000 by 2040 in England alone.^2^ However, the period before the end of life brings its share of challenges for the person living with dementia and their families. As dementia progresses, people may struggle to communicate their symptoms and concerns due to declining cognition. This may leave problems undetected and undertreated in the last months and years of life, causing distressing symptoms and jeopardized quality of life.^3^

Person‐centered care is a cornerstone of dementia care that seeks to deliver care aligned with individual priorities and preferences.^4^ Communication and shared decision‐making are key components of person‐centered care^5^ to assess symptoms and care priorities, agree and review care and treatment plans.^6^, ^7^ For care to be person‐centered, it is essential for the person with dementia to be involved in the decision‐making process regarding their care and treatment.^8^, ^9^, ^10^ Poor communication between the person with dementia and their families and health care practitioners compromises the experiences and outcomes of care for the person with dementia nearing end of life. The National Institute for Health and Care Excellence (NICE) defines shared decision‐making as “…when health professionals and patients work together. This puts people at the center of decisions about their own care and treatment.”^11^ Shared decision‐making in clinical practice involves providing information and supporting the person through consideration of available options to make informed decisions regarding care and treatment.^12^

Person‐centered outcome measures (PCOMs) are powerful tools in care delivery that facilitate and promote better communication between individuals and professionals. PCOMs include patient‐reported outcome measures (PROMs) and proxy‐reported outcome measures. PROMs are completed by the individual to measure perceptions of health, whereas proxy outcome measures are completed by families and professionals on behalf of the individual when unable to complete by themselves, such as in advanced disease.^13^ However, the focus remains on matters and preferences important to individuals. Systematic reviews on the use of PCOMs in routine care for patients with chronic progressive conditions have demonstrated that this can improve practitioners’ identification of individual needs and priorities, support shared decision‐making between the practitioner and patient, and in turn improve management of distressing symptoms and concerns, and health‐related outcomes of care.^13^, ^14^ However, little is known about using PCOMs for people with dementia to optimize shared decision‐making and improve outcomes of care. This review aimed to explore how PCOM use in routine dementia care facilitates shared decision‐making and improves outcomes of care. The findings inform a logic model depicting the processes to use a PCOM in clinical care and how this could improve outcomes of care.

## 2 METHODS

### 2.1 Study design

This systematic review drew on the guidance of Pope et al. for the conduct of narrative synthesis,^15^ and is reported in accordance with the Preferred Reporting Items for Systematic Reviews and Meta‐Analysis (PRISMA).^16^ A protocol for the review was registered on PROSPERO (ID: CRD42020189292).

**RESEARCH IN CONTEXT**

1. **Systematic Review**: We searched PsycINFO, MEDLINE, CINAHL, and ASSIA from databases and included studies reporting experiences and/or impact of person‐centered outcome measure (PCOM) use among people with dementia, family carers, and/or practitioners.
2. **Interpretation**: This systematic review reports on how PCOMs can enable shared decision‐making between people with dementia, their family carer, and health and social care professionals, and improve outcomes of care for people with dementia. We found that the use of PCOMs in routine care can facilitate shared decision‐making through “knowing the person,” “identifying problems, priorities for care and treatment and goal setting,” and “evaluating decisions.” Family carers are essential to uphold the priorities for the person with dementia in decision‐making about care and treatment.
3. **Future Directions**: Further research is required to understand the use of PCOMs with people living with dementia at home to improve decision‐making and outcomes of care, and training for family carers to use PCOMs.

### 2.2 Theoretical underpinning

The model of Groen Van de Ven of collaborative deliberation in dementia care networks underpins this review's understanding of shared decision‐making.^17^ This model comprises seven important elements to shared decision‐making, such as involving the network of an individual when decisions are to be made. This network involves the individual themselves, and their family carers. We described in **Table** S1 each element of the model and how we used them in our review process, such as abstraction, analysis, and interpretation of review data.

### 2.3 Search strategy

A Population, Intervention, Comparator, Outcome, Study type (PICOS) analysis was undertaken to develop and structure the search strategy.^18^ Search terms were informed by earlier systematic reviews^13^, ^19^, ^20^, ^21^ and refined with an information specialist and co‐authors (C.E.S. and C.E.). Four databases were searched from their inception (1806) to July 21, 2020: PsycINFO, MEDLINE, Cumulative Index to Nursing and Allied Health Literature (CINAHL), and Applied Social Science Index and Abstract (ASSIA). **Table** S2 presents the search strategy. We also reviewed PsycExtra database to identify gray literature such as quality improvement studies. Citation tracking and reference chaining were used to supplement the electronic searches.

### 2.4 Eligibility criteria and study selection

Studies of any design that used quantitative, qualitative, or mixed methods to report on the experience and impact of PCOM use among people with dementia, their family carer, and health or social care practitioner were included. Studies of any assessment measurement tool used in routine care, such as a pain assessment tool,^22^ were included to understand how a wide range of outcome measures are used to support shared decision‐making. PCOMs could be symptom specific, such as pain, or encompass multiple health domains. Process outcomes included any shared decision‐making element, as defined by Groen Van de Ven et al.,^17^ or communication. We included verbal communication exchange that occurred between the person and or their family carer, and care professionals. Outcomes of care included quality of life, as defined by the World Health Organization (WHO),^23^ function, and well‐being (Table 1).

**TABLE 1:** Eligibility criteria

**Criteria**	**Inclusion criteria**	Exclusion criteria
**Population**	Dementia/cognitive disorder/cognitive impairment related to dementia, carers, and care professionals	Cognitive impairment that is not dementia, for example, depression
**Intervention**	Person‐centered outcome measures (PCOMs) or assessment measure, that is, patient‐ or proxy‐reported outcome measures, and assessment measures that are person centered in natured and designed to improve care/outcomes for the person with dementia, such as dementia‐care mapping. Single item/multi‐domain	Outcome and assessment measures not person centered in nature or not focused on improving care, for example, diagnostic
**Outcome**	**Process outcomes**: Shared decision‐making, communication, person‐centered care	Diagnostic, for example, to diagnose dementia
	**Outcomes of care** Quality of life outcomes Daily living activities Function Physical and psychological well‐being, for example, agitation.	
**Comparator**	Any comparator ‐ Usual care, other interventions, or no comparators	
**Study design**	Quantitative, qualitative, and mixed methods studies	Case studies, non‐primary studies, for example, systematic review, focused solely on the development of outcome measures/testing psychometric properties/not how outcome measure is used in routine care.

One reviewer (J.A.) screened all titles and abstracts using the Covidence platform (https://www.covidence.org/). The full texts of eligible articles were screened independently by two reviewers: J.A. and one other (J.G., M.R., L.C., or E.Y.). Agreement between independent reviewers of full text screening was 77%. Any discrepancies were discussed between reviewers, and where disagreements remained, other reviewers were consulted (C.E.S. and C.E.). Eligibility of the non–English‐language papers were reviewed by researchers who spoke the language (including, Spanish, Mandarin, and German). We did not identify any other non–English‐language papers.

### 2.5 Data extraction and management

A template was used to extract and capture data from each study including its design, sample and setting, the PCOMs used, shared decision‐making elements, and outcomes.^18^ We extracted the effect of the intervention on process and quality of life outcomes. Data from all included studies were used to populate the logic model, including context, intervention, and outcomes. We extracted the narrative/qualitative and statistical significance (if available) of the effect of the intervention on process and quality of life outcomes. The collaborative deliberation model of Groen Van de Ven^17^ guided the identification and extraction of shared decision‐making elements. Data extraction was completed by one reviewer (J.A.), with 50% checked by a second reviewer (J.G.).

### 2.6 Quality appraisal

J.A. evaluated research studies for methodological quality using the Mixed‐Methods Appraisal Tool (MMAT), with 50% checked by J.G. to assure consistent appraisals. The MMAT contains five quality criteria that are rated as “Yes,” “No,” or “Can't tell,” and each study scores from 1 to 5, with higher scores indicating higher quality.^24^ The quality improvement studies were appraised using the Quality Improvement Minimum Quality Criteria Set (QI‐MQCS),^25^ which includes 16 domains that are rated as “met” or “not met.” Although, the QI‐MQCS does give quality threshold scores, other researchers have interpreted scores of >15 items “met” as perfect quality, >12 as good, >9 as moderate, and ≤9 as insufficient quality.^26^ We did not exclude studies based on quality rating; rather this was used to identify areas of uncertainty in the findings and logic model, such as components with low quality evidence.

### 2.7 Data analysis

We used tabulation and grouping techniques to inform a narrative summary of the data.^15^ Information on context, PCOMs’ components, and shared decision‐making were tabulated. Data on shared decision‐making and delivery were transferred to NVivo 12 for analysis.^27^ Data were inductively analyzed by J.A. and discussed with C.E. and C.E.S. to identify delivery themes and implementation requirements. Shared decision‐making data were coded and deductively categorized into themes informed by the model of shared decision‐making of Groen van de Ven et al.^17^. Areas of similarity and divergence across shared decision‐making elements were explored. Quantitative data were collated and narratively summarized, such as outcomes.

We used the results to develop a logic model (a diagrammatic representation) of how PCOMs facilitate shared decision‐making.^28^ We populated a logic model template with the extracted data from included studies, such as setting, population, PCOM components, and outcomes. The model describes the anticipated delivery mechanisms, intervention components, mechanisms of impact, and intended outcomes.

## 3 RESULTS

### 3.1 Study selection

**FIGURE 1:**
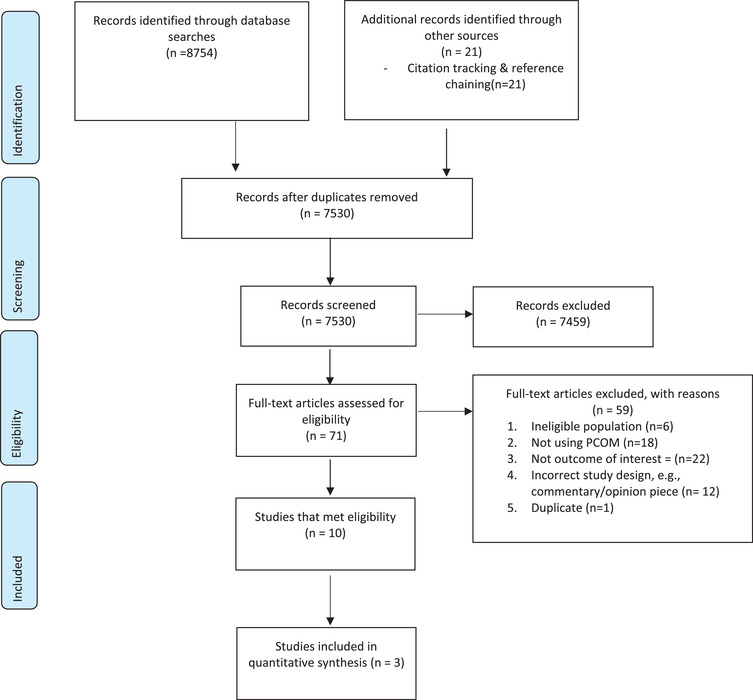
PRISMA flow diagram

Ten studies reported in 12 articles met the review's eligibility criteria (see **Figure** **1**). We identified no eligible gray literature.

### 3.2 Study characteristics

**Table** **1** presents study characteristics. Sample sizes ranged from 10 to 429 and totaled 1064 participants across the 10 studies (12 articles), comprising people with dementia, family carers, or health and social care practitioners. Qualitative studies included in this review used focus groups,^29^ interviews,^30^ or observations.^31^ Others were feasibility studies (*n* = 2),^32^, ^33^ pilot study,^34^ pilot randomized controlled trial (*n* = 1),^35^ quasi‐experiment (*n* = 1),^36^ and quality improvement (*n* = 2).^37^, ^38^ Studies originated from the United States,^30^, ^35^, ^37^Canada,^33^ Europe,^31^, ^32^, ^36^, ^38^, ^39^ and Australia.^29^, ^34^, ^40^ Half of the studies were conducted in long‐term care settings (*n* = 5), such as nursing homes.^31^, ^32^, ^33^, ^36^, ^37^ The remainder were based in a mixed setting,^38^ community,^34^ emergency department,^29^, ^30^ and a geriatric/primary care clinic.^35^ Health‐related outcomes measured by the studies included function and activities of daily living^33^ and palliative care needs (symptom assessment).^36^ Process outcomes included person‐centered communication.^35^ (**Table** **2**) None of the studies reported any adverse events or unintended consequences of using PCOMs on patient outcomes. There were no differences in the elements of shared decision‐making achieved when using a multi‐domain PCOM compared with symptom‐specific PCOM.

**TABLE 2:** Study characteristics and quality appraisal

**First year, Author**	**Study design,**	**Population, setting,** **Aim**	**Sample size**	**MMAT score, QI‐MQCS**	**Results (impact of PCOMs use on care)**
**Fry et al. (2017),** ^29^ **Australia**	Focus groups	Dementia, *Emergency department* To explore emergency nurses’ perception of the feasibility of the PAINAD tool in people with cognitive impairment	36	Qualitative ‐ *****	PAINAD gives structure to pain assessment. PAINAD assists to convey pain intensity
**Moore and Sullivan (2017),** ^30^ **USA**	Qualitative interviews	Nursing home residents including people with dementia, *Emergency department* To describe the results from focus groups meetings aimed enhancing a tool for care transitions for individual with dementia and determining the need for such a tool	26	Qualitative ‐ ****	The ADMIT Me tool has the potential to significantly impact communication and collaboration. The ADMIT Me tool brings attention to behavioral concerns and address techniques; it allows the nurse to create an individualized plan of care and provide patient‐centred care
**Holle et al. (2015),** ^31^ **Germany**	Qualitative interviews/observation	Dementia, *Nursing/care home/ special care unit* To explore nursing staff's experience with a dementia specific case conference concept in combination with the innovative dementia‐oriented Assessment tool (IDA)	84	Qualitative ‐ *****	CC‐IdA helpful for handling of challenging behavior, changes in communication with residents and triggers of challenging behavior. Barriers to implementation of the tool includes lack of moderation skills, limited dementia knowledge, lack of patient information, and little involvement from other care professionals.
**Ellis‐Smith et al. (2018),** ^32^ **UK**	Feasibility study and process evaluation	Care home residents (dementia), *Residential care home* To explore the mechanisms of action, feasibility, acceptability and implementation requirements of a measure, the Integrated Palliative care Outcome Scale (IPOS‐Dem),	26	Qualitative ‐ *****	IPOS‐Dem improved observation and awareness. Collaborative assessment, comprehensive picture of person, systemic record keeping, improved monitoring and review. Potential to: improve symptom management, to facilitate early symptom detection and problems, comprehensively address care needs, and increase family empowerment and engagement in care.
**Hartman et al. (1997),** ^33^ **Canada**	Feasibility study, pre‐post test	Dementia, *Nursing/care home/ special care unit* To report on the feasibility and responsiveness of GAS in an SCU	10	Quantitative ‐ **	The mean GAS admission, follow‐up and change scores (SD) were as follows: assessment: 35.4 ± 7.7, follow‐up: 53.4 ± 16.0, change: 18.0 ± 19.6. The paired *t*‐statistic was 2.9 (df = 9, *p* = .017). The effect size statistic was 2.34; Cohen's d considers effect sizes greater than .80 to be large. Norman's responsiveness statistic was .43.
**Hermans et al. (2018),** ^36^ ***Hermans et al. (2014)*,** ^39^ ***protocol paper)*, Belgium**	Quasi‐experimental, pretest–posttest study	Dementia, *Nursing home* To evaluate whether using the interrail Palliative Care instrument (the interrail PC) in nursing home is associated with reduced needs and symptoms in residents nearing end of their lives	429	Quantitative ‐ ****	No significant difference between the post‐test POS scores of the control and intervention nursing home residents. Post‐test POS scores in the intervention nursing homes (*n* = 12) were significantly higher on item 9 (“wasted time”), POS score of 0.15 (CI 95% = 0.05–0.25; *p* = .004).
**Meyer et al. (2019a),** ^34^ **Meyer et al. (2019b),** ^40^ **Australia**	Pilot study	Dementia, *Community* To outline the development of a discussion tool based on decision aid principles, designed for use by community service providers to provide choices for people with dementia and their caregivers in addressing high falls risk factors.	25	Quantitative ‐ **	After implementation of the discussion tool (FROP‐Com), there was better collaboration between people with dementia, their family carers and care professionals and uptake in evidence‐based falls prevention strategies.
**Wolff et al. (2018),** ^35^ **USA**	Randomized controlled pilot trial	Patients with cognitive impairment or dementia*, General primary care clinic and geriatric clinic* To examine whether a patient‐family agenda setting interventions improves primary care visit communication for patients with cognitive impairment	93	Quantitative ‐ ****	For the primary outcome of patient‐centred communication, communication was more patient‐centred in the intervention dyads visits compared to control (ratio of 0.86 vs. 0. 68; *p* = .046), using the Patient‐Family Agenda Setting Checklist and audio recording of patient visitation For the secondary outcome of verbal, intervention companions were more verbally active compared to control) at the two general clinics (21.3% vs. 16.1% of visit statements at clinic 1; *p* = .005 and 21.5% versus 15.8% at clinic 2; *p* < .001).
**Kinley et al. (2019),** ^35^ **UK**	Audit	Dementia, *Community and inpatient services* To understand how best to implement outcome measures into services for people with dementia across clinical settings	225	Audit – insufficient quality	Benefits of using outcome measures included: Promoting a comprehensive assessment, the identification of symptoms/problems and requirement to address (such as seeking GP's advice). People with dementia enjoyed the opportunity to discuss problems/concerns/care/treatment.
**Dahl et al. (2008),** ^37^ **USA**	Quality improvement	Dementia, *Dementia care facility* (a) To obtain updated and timely information from the family and nursing staff on present and past behavioral problems in long‐term care residents with difficult behaviors, (b) to determine whether currently prescribed psychotropic drugs have been useful and if not, appropriately tapered, (c) to document the presence of possible adverse drug events associated with atypical psychotropic drug use, (d) to provide the family or responsible party with updated information on the risk‐benefit ratio of antipsychotic drug use, and (e) provide updated clinical information to the pharmacist and physician of record to inform continued pharmacological management.	110	Quality improvement – Moderate quality	After using PAT there was 1.5% ↓ in number of residents prescribed antipsychotic medication. Antidepressant use remained the same. 0.8% ↑ in hypnotics use. 7.3% ↓ in ACE inhibitors. 1.2% ↓ in Namenda

ADMIT Me Tool, Alzheimer's Dementia Memory Impaired Transitions; CC‐IDA, Dementia specific case conferences with the Innovative dementia oriented tool; GAS, goal attainment scale; MMAT, Mixed‐Methods Appraisal Tool; PAINAD, The Pain Assessment in Advanced Dementia Tool; POS, Palliative Outcome Scale; QI‐MQCS, Quality Improvement Minimum Quality Criteria scored as >15 items ranked as “met” as perfect quality, >12 as good, >9 as moderate, and ≤9 as insufficient quality.

*Represents the number of quality appraisal criteria a study has met, for example, if a study has **, then two of five categories met.

### 3.3 Quality appraisal

All included studies were assessed for quality, of which six met four or more of five criteria, indicating high quality,^29^, ^30^, ^31^, ^32^, ^35^, ^36^ such as using the appropriate qualitative approach to answer the research question, and a clear research question. Two studies met two of the five criteria.^33^, ^34^ An example of criterion not met includes confounders not being accounted for in design and analysis of the studies. The quality improvement study was determined to be of moderate quality because 10 of 16 categories met the minimum quality criteria standard,^37^ whereas the audit study met six categories,^38^ indicating insufficient quality. Full appraisals are provided in the Supplementary Material.

### 3.4 PCOM characteristics and relationship to shared decision‐making

**Table** **2** describes the characteristics of the PCOMs that focused on symptom assessment (*n* = 5), medication management (*n* = 1), falls prevention (*n* = 1), pain management (*n* = 1), goal setting for function and activities of daily living (*n* = 1), and patient‐centered communication (*n* = 1). Six were multi‐domain in nature. The PCOMs were administered mainly by the care staff (*n* = 6) or research staff (*n* = 2). Time to completion ranged from less than 10 minutes to 2 hours (during multidisciplinary meeting where multiple residents were discussed).^31^, ^32^, ^33^, ^35^

The most commonly reported element of shared decision‐making was “constructive network engagement,” which was evident in all studies, and “Recognizing the need for a decision now,” which was present in four studies.^29^, ^34^, ^35^, ^38^ There was less evidence for the remaining shared decision‐making elements (**Table** **4**).

### 3.5 Themes

From the narrative synthesis, we identified four overarching themes about how PCOMs may enable shared decision‐making, including: “knowing the person,” “identifying problems, priorities for care and treatment, and goal setting,” “evaluating decisions,” and “implementation considerations for PCOM use.”

#### 3.5.1 Knowing the person

This theme reflects the element “constructive network engagement” in the model of Groen Van de Ven^17^ and involved the network of the person with dementia, including the person with dementia when able, the family carers, and the care professionals **(see Table** S4). Family involvement was crucial in the process of shared decision‐making to complete a comprehensive assessment encompassing multiple health domains and to identify symptoms and priorities for the person with dementia, particularly when no longer able to express preferences.^30^, ^31^, ^32^, ^33^, ^34^, ^35^, ^36^, ^37^, ^38^ The use of a PCOM to assess symptoms created an opportunity for practitioners to engage the person with dementia (when able) and their family to obtain accurate and up‐to‐date information about the person's symptoms and concerns.^29^, ^32^, ^33^, ^34^, ^35^, ^37^, ^38^ Use of PCOMs increased knowledge and understanding of the person's symptoms and concerns. Using a PCOM formed a structure for practitioners to involve the family in assessments and review of care and treatment, and work collaboratively by discussing concerns identified.^30^, ^31^, ^36^

“The Psychotropic Assessment Tool (PAT) questionnaire is filled out bi‐yearly and on an as‐needed basis on all patients at two of the quarterly family bi‐yearly and on an as‐needed basis on all patients at two of the quarterly family conference meetings.”^37^

#### 3.5.2 Identifying problems, priorities for care and treatment, and goal setting

Elements of shared decision‐making in this theme were “recognizing the need for a decision now,” “defining what needs to be decided on,” “developing alternatives and constructing preferences through deliberation,” and “trying out alternatives” (see Table S4). Family carers continued to be involved in the process of shared decision‐making with this stage involving discussing symptoms and concerns, identifying care and treatment priorities for the person with dementia and their family, and setting goals of care.

“Recognizing the need for a decision now” concerned the network of the person with dementia using a PCOM to identify distressing symptoms, and then working with the person with dementia and family carer to prioritize concern and the plan and goals of care.^29^, ^34^, ^35^, ^38^ “Defining what needs to be decided on” involved goal setting in relation to the symptoms and concerns identified. Goals were set to align with the identified priorities and preferences for the person with dementia and/or their family carer.^33^, ^34^, ^35^ For example:

“Rather than planning global activity programs, emphasis was shifted to a thorough assessment of each individual's skills as well as family/resident involvement in identifying personal interests and goals.” ^33^

Two elements of shared decision‐making were combined as they overlapped, including “developing alternatives” and “constructing preferences through deliberation and trying alternatives.” The PCOM was used as the basis to discuss the symptoms and options for care and treatment, and to prioritize preferences.^34^ One study identified that a PCOM is useful in multidisciplinary meetings if the meeting is structured and focused on the priorities for care of the person with dementia.^31^

#### 3.5.3 Evaluating decisions

This element of shared decision‐making is “evaluating decision‐making” and involved using PCOMs to support discussions and review decisions made about the care and treatment and outcomes of care (see Table S4). This identified opportunities to review the plan of care to ensure keeping up with changes in symptoms and concerns. For example:

“Based on the CAPs results from the interRAI PC assessment and the accompanying manuals, care professionals were able to evaluate, adapt, and design individual care plans.”^36^

#### 3.5.4 Implementation of PCOMs to enable shared decision‐making

Important implementation characteristics for PCOMs included “ease of use” of the intervention, “availability of a manual” detailing how to use the PCOM, and “availability of technology” to complete and interpret the PCOM, leading to improved monitoring care management.

One study in care homes reported that technology could support PCOM use with a web‐based version of the PCOM, which enabled family members to access the PCOM remotely and discuss with care professionals when visiting or by phone.^32^

“Touchscreen technology, while not essential, was identified as a potential key facilitator in completing IPOS‐Dem, storing records, monitoring over time, and communication including online access for family members.”^32^

The PCOM should be easy to understand and quick to complete without specialist qualification such as a registered nurse.^32^ The main challenges associated with using PCOMs included staff's lack of knowledge about the PCOM and knowledge to interpret the results to inform the assessment and decision‐making about care and treatment.^31^ Using a PCOM to assess pain for a person with dementia required nurses’ expertise to identify discomfort and pain, assess, manage, and monitor the outcome after intervention.^29^ Manuals were identified as an important way to provide training about how to use the PCOM intervention in routine care, such as frequency, and how to interpret the measurement of symptoms and change overtime to inform clinical decision‐making about care and treatment.^30^, ^32^

Important requirements to use PCOMs for shared decision‐making included leadership and organizational support, but “staff busyness, heavy workload, and time constraints” contributed towards the likliehood of the PCOM being used to support identification of distressing symptoms.^31^ ^,^ ^32^ Despite time constraints, staff with a positive attitude toward the intervention supported adoption.^30^ Staff understanding the purpose of the PCOM and potential benefits for patients was vital to support staff use.

“Leadership was seen as required to support adoption by all care home staff, ensuring that care home staff remember to use the measure, and ensuring they understand its purpose; thus ensuring that the measure is recognized as a valued tool to support care provision despite additional time burden.”^32^

### 3.6 Outcomes measured and intended benefits

Three studies measured outcomes for the person with dementia (**Table** **3**). These concerned physical function and activities of daily living (*n* = 10),^33^ palliative care needs (*n* = 429),^36^ and patient‐centered communication (*n* = 93).^35^ Patient‐centered communication was determined by a validated system that described the frequency of communication relating to psychosocial and socioemotional topic.^35^ One study reported the use of a PCOM to manage medications by facilitating a twice‐yearly review between health and social care professionals and family carers.^37^ The intended benefit of using PCOMs varied and included symptom assessment, such as neuropsychiatric symptoms^31^; physical symptoms, such as pain^29^; and comprehensive assessment across health domains.^30^, ^32^, ^36^, ^38^ An example is the Integrated Palliative care Outcome Scale for Dementia (IPOS‐Dem) that assesses multiple health domains, such as spiritual, psychosocial, and physical.^32^ One study focused on using PCOMs to assess, identify, and monitor risks of falls^34^ by ranking fall‐risk factors and using the PCOM as a discussion tool to develop strategies to manage and reduce risk of falls. One study focused on improving communication between the person with dementia and the family carer and care professionals about their concerns and priorities for care and treatment.^35^

**TABLE 3:** PCOM description and identified mechanisms of impact

**Author (year), country**	**PCOM name**	**PCOM focus**	**Intervention implementation**	**Who administered the PCOM and how**	**Who was involved in the process of SDM?**	**Outcome measured/impact on care**	**Mechanisms of impact on outcomes of care**
**Fry et al. (2017),** ^29^ **Australia**	PAINAD	Pain management	Not stated	Nurses Format: not stated	Family carer and care staff	PAINAD conveys pain intensity	1. Collaboration with family to complete pain assessment if available (*constructive network engagement*) 2. Family carer able to raise awareness in medication needs as they are able to detect changes or improvements in pain level due to time spent with the person with dementia (recognizing the need for a decision now)
**Moore and Sullivan (2017),** ^30^ **USA**	Alzheimer's, Dementia, Memory, Impaired Transitions (ADMIT Me tool)	Symptom assessment	Not stated	Care staff Format: not stated	Family carer and HCP	The ADMIT Me Tool impacts on communication and collaboration	1. The ADMIT Me tool allowed health care professionals to be up to date with the patient, and also discussion between care professionals and families about the patient's conditions. 2. Identification of medical and behavioral problems and understanding of the person
**Holle et al. (2015),** ^31^ **Germany**	Dementia‐specific case conferences with the innovative dementia‐oriented assessment tool (CC‐IA)	Neuropsychiatric symptom assessment	90–120 minutes during monthly case conferences	Nursing staff Format: not stated	Family carer and care professionals	The CC‐IdA used to support manage challenging behavior changes in residents	1. CC‐IdA creates an opportunity to collaborate with family to interpret results of the assessment (constructive network engagement)
**Ellis‐Smith et al. (2018),** ^32^ **UK**	Integrated Palliative Outcome Scale Dementia (IPOS‐Dem)	Symptom assessment	The mean time it took to complete IPOS‐Dem at baseline was 8.48 minutes (SD 4.98) and at final time point was 5.60 minutes (SD 1.45).	Face to face by care home staff who administered the IPOS‐Dem to all participating residents at baseline and at 12 weeks. Format: paper	Family carer and HCPs	The IPOS‐Dem was acceptable and feasible for use in routine care to support the comprehensive assessment of symptoms and concerns of care home residents and their family members	1. Use of IPOS‐Dem created an opportunity for collaborative assessment between family and care home staff, 2. Improved communication between family and staff 3. Increased family empowerment and engagement in care 4. Improved observation and awareness of symptoms and concerns 5. Care planning and changes to care provision Improved monitoring and review
**Hartman et al. (1997),** ^33^ **Canada**	Goal Attainment Scale (GAS)	Function and therapeutic recreations	A GAS process was followed for 10 residents of a 30‐bed SCU for persons with dementia. One or two goals were identified for each resident; an average of 20 minutes was required to construct a GAS follow‐up guide.	Face to face Staff (occupation therapist, the therapeutic recreation specialist and occupational therapy assistant). Format: not stated	Person with dementia, family carer and HCP	Function and activities of daily living	1. Use of GAS scale creates an opportunity to involve the family to support completion, thus leading to a comprehensive assessment to understand the function and activities of interest to the person with dementia (*constructive network engagement*) Allowed opportunity for specific and personalized goal setting
**Hermans et al. (2018),** ^36^ ***Hermans et al. (2014)*,** ^39^ ***protocol paper)*, Belgium**	InterRAI palliative care (InterRAI PC)	Symptom assessment	Over the course of the year, these care professionals filled out the InterRAI PC every 3 months for all residents identified as eligible. Training on PCOM provided	Care staff (manager) Format: not stated	Person with dementia, family carer and HCP	Palliative Care needs	1. The use of the InterRAI. Created opportunity for collaboration between staff and family and PwD (constructive network engagement) 2. Development/adaptation of care plans (*evaluating decision‐making*)
**Meyer et al. (2019a),** ^40^ **Meyer et al. (2019b),** ^34^ **Australia**	Falls Risk for Older People‐Community (FROP‐Com)	Falls prevention	Need for a knowledge broker who is responsible for directing the discussion using motivational interviewing techniques and clinical judgement. This is likely a care staff	Care staff Format: not stated	Person with dementia, family carer and HCP	Increased uptake of falls‐prevention strategy	1. Collaboration between people with dementia, their caregivers, and health professionals (*constructive network engagement*) 2. Gave participant dyad the opportunity to identify high‐risk factors that are a priority for them through discussion with a “knowledge broker” (*recognizing the need for a decision now*) 3. Allowed for specific and personalized goal setting (*defining what to decide on*) 4. Allowed for development around alternative falls strategies through discussion of pros and cons 5. Allowed for development of preferences 6. Allowed for evaluation of decision‐making
**Wolff et al. (2018),** ^35^ **USA**	Agenda checklist	Patient centred communication	One‐page checklist completed in the waiting room by older adults and their family companion; 69.4% completed the measure in ≤10 min, 26.5% completed in ≤15 min, and 4.1% completed it in ≥15 min	Research team Format: paper	Person with dementia and carer as a dyad; then discussed with care professional	Improved communication	1. Person with dementia and their family carer able to discuss the role of the carer during the visit, using the agenda checklist (*constructive network engagemen*t) 2. The person with dementia and their carer identifies health priority and what they want to discuss with the clinician together (*recognizing the need for a decision now*)
**Kinley et al. (2019),** ^38^ **UK**	IPOS‐Dem, Austrailia‐modified, Karnofski Performance Scale (AKPS), palliative care phase of illness	Symptom assessment/function and dementia severity	Face to face by clinician team Face to face training for staff	Care staff Format: paper	Person with dementia, care staff, and family carer	Promoted comprehensive assessment	1. The PCOMs allowed family members and person with dementia to contribute toward assessment process by completing the outcome measure or contributing, and having discussions around care and treatment (*constructive network engagement*) 2. People with dementia enjoyed being able to contribute and discuss their concerns, needs, and treatment 3. Increased awareness of problems by care staff and family members not previously identified
**Dahl et al. (2008),** ^37^ **USA**	The Psychotropic Assessment Tool (PAT)	Medication management	The PAT questionnaire is filled out bi‐yearly and on an as‐needed basis on all patients at two of the quarterly family conference meetings. The family conference meeting involves the interdisciplinary staff, which consists of a family member (attends 60%–70% of time) Format: not stated	Face to face with Medical staff (Multidisciplinary team) Format: not stated	Family carer and HCP	Better medication management	1. The PAT creates an opportunity for collaboration between families and care professionals. 2. Completion of PAT led to “PAT chat,” which facilitated conversation between family and MDT team to allow for comprehensive picture and assessment (*constructive network engagement*) and construction of preferences through deliberation and trying out alternatives

ADMIT Me Tool, Alzheimer's, Dementia, Memory, Impaired Transitions; CC‐IDA, Dementia specific‐case conferences with the innovative dementia‐oriented assessment tool; FROP‐Com, Falls Risk for Older People‐Community; GAS, Goal Attainment Scale; InterRAI PC, InterRAI palliative care; IPOS‐Dem, Integrated Palliative Outcome Scale for Dementia; PAINAD, Pain assessment in advanced dementia; PAT, Psychotropic Assessment Tool; PCOM, Person centred outcome measure; SDM, Shared decision‐making.

Sections in parentheses and italics are components of the underpinning shared decision‐making model.

**TABLE 4:** Elements of shared decision‐making present in each study

	**First author (year)**									
**Shared decision‐making elements** ^17^	**Dahl et al. (2008),** ^37^ **USA**	**Hartman et al. (1997),** ^33^ **Canada**	**Ellis‐Smith et al. (2018),** ^32^ **UK**	**Hermans et al. (2018),** ^36^	**Holle et al. (2015),** ^31^ **Germany**	**Moore and Sullivan (2017),** ^30^ **USA**	**Kinley et al. (2019),** ^38^ **UK**	**Meyer et al. (2019b),** ^34^	**Fry et al. (2017),** ^29^ **Australia**	**Wolff et al. (2018),** ^35^ **USA**
Constructive network engagement	✓	✓	✓	✓	✓	✓	✓	✓	✓	✓
Recognizing the need for a decision now							✓	✓	✓	✓
Defining what to decide on		✓						✓		✓
Developing alternatives								✓		
Constructing preferences by deliberation and trying out alternatives								✓		
Evaluating decision‐making	✓			✓						
Integration of multiple preferences										

Table 4 shows the components of shared decision‐making within the articles included in this systematic review.

## 4 DISCUSSION

**FIGURE 2:**
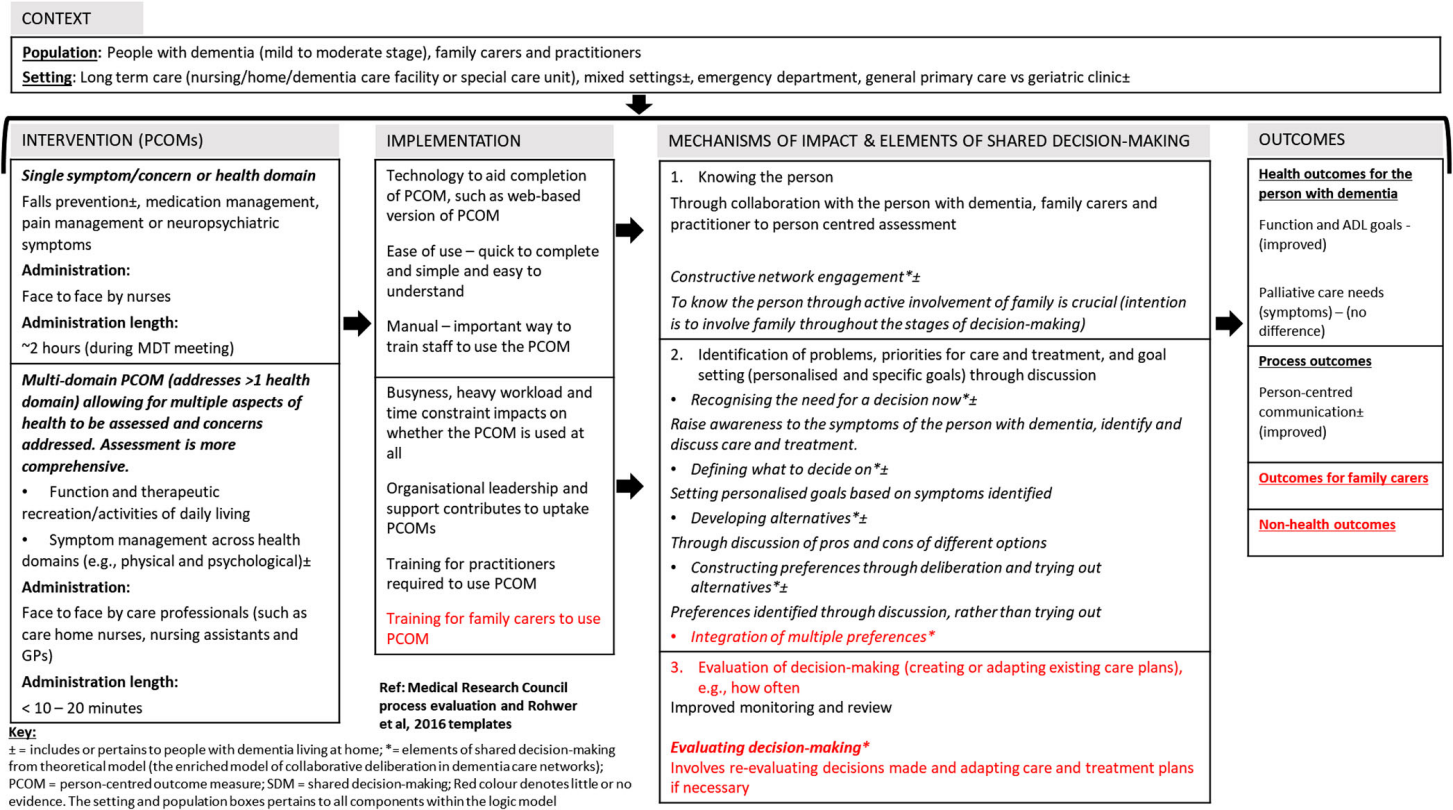
Logic model of person‐centered outcome measure (PCOM) use to enable shared decision‐making in the routine care of people with dementia.

To our knowledge, this is the first systematic review to explore how the use of PCOMs could enable shared decision‐making in the routine care of people with dementia and improve care outcomes. There was evidence that PCOMs can enable shared decision‐making, through “knowing the person,” “identifying problems, priorities for care and treatment and goal setting,” and “evaluating decision‐making.” “Constructive network engagement” was the most common element of shared decision‐making. There was little evidence for the remaining five elements of shared decision‐making, and none for integrating multiple preferences. However, it is important to note that PCOMs were not created to specifically enable shared decision‐making. This may explain these gaps in the evidence base. Limited evidence from two studies linked PCOM use to improved patient outcomes, in terms of person‐centered communication^35^ and physical function and activities of daily living.^33^ A third study reported no difference in identification of palliative care needs using a PCOM compared with care as usual.^36^ Requirements to use PCOMs included staff expertise and associated training, and ease of use in routine care to minimize the demands on staff time The findings informed a logic model on how PCOMs could enable shared decision‐making detailing the components, implementation processes, mechanisms of impact, and linkages with outcomes (see **Figure** **2**).

### 4.1 The logic model of how PCOMs enable shared decision‐making

The logic model shown in **Figure** **2** illustrates linkages of key PCOM mechanisms with respective elements of shared decision‐making. These include increased knowledge about the person with dementia through undertaking a person‐centered assessment involving the person, their family carer, and care professional. PCOMs allowed collaborative opportunities to better know the person with dementia and their families. This collaboration reflects the element of “constructive network engagement.” PCOMs also enabled the identification and discussion of symptoms and concerns, priorities for care, and goal setting and re‐evaluating the decisions made. Active involvement of the family carer and person with dementia led to improvements in patient‐centered communication, physical function, and activities of daily living. The elements of shared‐decision making were similar in a multi‐domain PCOM compared with a single symptom/concern/health domain PCOM. This would appear to imply similar benefits in discussing care and treatments for care and improves outcomes of care. Our logic model identifies areas of uncertainty in using PCOMs such as training needs for family carers to discuss their concerns and priorities using PCOMs, and the potential benefit of working in this way, such as empowerment of family carers. The improvements shown in the outcomes of communication, function, and activities of daily living corroborate evidence from other progressive conditions, such as cancer, that using PCOMs in routine care can improve communication between the patient and practitioner, and aspects of quality of life, such as emotional and psychological well‐being.^13^, ^14^, ^41^

### 4.2 Importance of constructive network engagement in shared decision‐making

Involvement of the family carer was identified as crucial to support shared decision‐making. This stresses the importance of relational dementia care that requires the family carer and health care professional to work together to facilitate goal concordant care, for care and treatment to align with the person's priorities and preferences.^42^ The involvement of family carers is a well‐reported key component of dementia care. Family carers of people with dementia provide support, are involved in various aspects of the person's care,^43^, ^44^ and are relied on to uphold the personhood of the person with dementia as the condition progresses.^45^ Family carers provide the majority of care for the person with dementia,^46^ and are at risk of carer burden, such as anxiety and physical ill health.^47^ It is, therefore, important to consider how shared decision‐making impacts family carer outcomes. An area of uncertainty in the logic model is how divergent preferences between the person and family carer may be managed, particularly when the person with dementia may lack insight into their condition. Where the person with dementia can contribute to decisions about care and treatment they may express different preferences and priorities to the family carer.^48^, ^49^ This shared decision‐making element of integration of multiple preference lacked evidence in this review. Furthermore, an evaluation of decisions made is important to ensure that care provision aligns with the priorities and goals discussed with the person with dementia and their family.

### 4.3 Importance of involving the person with dementia in shared decision‐making

This review recognized the importance of involving the person with dementia in the shared decision‐making process whenever possible. Some studies included people with dementia in a shared decision‐making process.^33^, ^34^, ^35^, ^36^ This aligns with person‐centered care of enabling the person with dementia to contribute to decisions about care and treatment, with involvement improving quality of life,^8^ particularly in the early stages of the disease.^8^, ^10^, ^50^

### 4.4 Limitations and future areas of research

Half of the studies included in this review were based in long‐term care, such as a care home. Only two studies included people with dementia living at home.^35^, ^38^ It is likely that different settings have different implications for PCOMs and their role in promoting shared decision‐making. Further research is needed to understand what is required to use a PCOM to enable shared decision‐making for people with dementia living at home and their family carers and care professionals, such as training. This would empower the family carer to use the PCOM to identify concerns and discuss priorities for care. There are individuals with no family members,^51^ but who may have close friends who act as their support; therefore the use of a PCOM could also be beneficial for such individuals. People with dementia living at home transition between care settings, particularly nearer to end of life.^52^, ^53^ Future research should explore how a PCOM could be used for people who may be transitioning between settings, such as from home to an acute hospital. PCOMs could support comprehensive assessment during transitions to know the person,^30^ enable discussion about their care priorities, and decide on care collaboratively within the care network. This could reduce unrecognized symptoms for the person with dementia, and working in this way may reduce risk of carer burden. People with early stage dementia may rely less on family carers to support decisions on care and treatment. However, many reported studies describing with people with early stage dementia were excluded at the full‐text screening stage as they did not include use of PCOMs in shared decision‐making. For example, publications focused on dementia care mapping, which did not detail assessment or shared decision‐making processes.^54^, ^55^ Only three studies quantitatively measured the use of PCOMs on process outcomes or outcomes of care, of which one was a pilot randomized control trial. The remaining were qualitative or audit studies.

Groen's Van De Ven's model of collaborative deliberation guided our understanding of shared decision‐making in dementia, and network involvement is a critical component. Despite our rigorous and systematic search for relevant articles, we found only three small trials with equivocal outcome data.^33^, ^35^, ^36^ This indicates that a research priority is for definitive trials to determine the effect of PCOMS on care and treatment outcomes. Our review findings would have also benefited from the inclusion of data on outcomes for family carers, who play a vital role in supporting the person with dementia. However, evidence of outcomes for family carers as a result of using PCOMs was lacking in this review, despite the wealth of research on interventions to improve outcomes for carers of people with dementia.^56^, ^57^ It is possible that the literature on PCOM use in dementia care focuses on benefits for people with dementia. We recognized that three of the included studies were of low to insufficient quality. We included these studies to learn about using PCOMs in routine care to enable shared decision‐making, as this is the first systematic review to our knowledge to explore this topic in dementia care. PCOMs are person‐centered assessments that help to understand symptoms and concerns and support conversations around those concerns and treatment options. However, they do not consider the values and habits of individuals. Our review could not explain how PCOMs would support non‐verbal communications or where individuals do not have capacity to make decisions.

### 4.5 CONCLUSIONS

This is the first review to consider how PCOMs could enable shared decision‐making in routine dementia care. The presentation of the findings as a logic model demonstrated how contexts, intervention components, implementation, and mechanisms can be linked to outcomes. Although, the evidence base is in a nascent stage of development, all studies showed at least one link between PCOMs and shared decision‐making, with evidence to suggest that using PCOMs could improve communication, function and activities of daily living. The findings indicate that the active involvement of a family carer and the person with dementia are key for the effective use of PCOMs to enable shared decision‐making. Further research is required to better understand the potential for PCOMs to improve health care outcomes for people with dementia through enhanced shared decision‐making, how to enable family carers to use PCOMs in discussion with practitioners, and how a PCOM can be used during transition in care settings.

## CONFLICT OF INTEREST

None declared. Author disclosures are available in the Supporting Information.

## AUTHOR CONTRIBUTIONS

Jesutofunmi Aworinde, Clare Ellis‐Smith, and Catherine J. Evans conceptualized the systematic review and developed the search terms and strategy. Jesutofunmi Aworinde screened all titles and abstracts. The full texts of eligible articles were screened independently by two reviewers: Jesutofunmi Aworinde and another (Juliet Gillam, Moïse Roche, Lucy Coombes, or Emel Yorganci). Any discrepancies were discussed between Jesutofunmi Aworinde, Clare Ellis‐Smith, and Catherine J. Evans. Studies eligible for data extraction were quality appraised by Jesutofunmi Aworinde and 50% by Juliet Gillam. Jesutofunmi Aworinde drafted the manuscript, which was reviewed by all.

## Supporting information



Supporting Information
