# Supplementary material for: How do person‐centered outcome measures enable shared decision‐making for people with dementia and family carers?—A systematic review
Source: Alzheimers Dement (N Y). 2022 Jun 6;8(1):e12304. doi: 10.1002/trc2.12304 (PMC9169867; doi:10.1002/trc2.12304)
Supplement: Supplementary file 1 — Supporting Information [file TRC2-8-e12304-s001.docx]

## Table S1: Application of shared decision-making model elements

| **Groen Ven de Ven model enriched model of collaborative deliberation (Groen van de Ven et al., 2017)** | **Application in this systematic review** |
| --- | --- |
| Constructive network engagement  “includes involving the network of the person with dementia and aligning to the nature of the longstanding family or spousal relationships in the network” | Any involvement of family carers, such as, to support assessment or discuss the symptoms of person with dementia identified from using a person-centred outcome measure |
| Recognizing the need for a decision now -  “negotiating with the network about emerging decision topics arising from the differences between participants in terms of recognising problems” | Discussion around what the problems are and what are the priorities to be addressed now |
| Defining what to decide on -  “explicating decision topics and the related goals, and choosing which problems need to be addressed now”. | Further discussion and clarifications around the problems and the goals the person and family want to achieve |
| Developing alternatives –  “includes developing alternatives together with the care network, often by trial and error. Care networks usually perceive a lack of readily available alternatives. Alternatives therefore depend on the possibilities and knowledge of the care network.” | Discussions around possible care and treatment options and deciding on an option(s) together within the network |
| Constructing preferences through deliberation and trying out –  “means combining and alternating deliberation and trying out alternatives to find out what suits best. The emotions and behaviours of people with dementia may be used by informal caregivers and professionals to interpret their preferences.” | Discussions around pros and cons of each treatment option and deciding together the best option(s) for the person with dementia |
| Multiple preferences integration –  “Means balancing the potentially conflicting interests and preferences of the person with dementia and the informal caregivers into a course of action.” | Management of multiple preferences for care and treatment that occur within the network, such as if the person with dementia prefers a different course of treatment or type of care to the family carer or care professional |
| Evaluating decision-making –  “is about looking back at decisions made and modifying decisions if necessary. It is also important to evaluate the process of reaching decisions in order to learn for future decision-making situations.” | This is about re-evaluating the decisions made at a later timepoint after the initial decision for care and treatment has been made. |

Note: This table demonstrates how we applied the elements of Elwyn’s model to understand, and analyse data around shared decision-making in this systematic review.

# Supplementary files

## Table S2 - search strategy

Database(s): **Ovid MEDLINE(R) ALL**1946 to July 21, 2020
Search Strategy:

| **#** | **Searches** | **Results** |
| --- | --- | --- |
| 1 | exp Dementia/ | 165350 |
| 2 | exp Cognition Disorders/ | 93778 |
| 3 | exp Alzheimer Disease/ | 93522 |
| 4 | exp care home/ | 47269 |
| 5 | exp nursing home/ | 9362 |
| 6 | exp assisted living facility/ | 1378 |
| 7 | (dementia or alzheimer* or cogniti* impairment or care home or nursing home or assisted living facilit*).mp. [mp=title, abstract, original title, name of substance word, subject heading word, floating sub-heading word, keyword heading word, organism supplementary concept word, protocol supplementary concept word, rare disease supplementary concept word, unique identifier, synonyms] | 290145 |
| 8 | exp Patient-Centered Care/ | 20199 |
| 9 | exp Process Assessment, Health Care/ | 4694 |
| 10 | exp Needs assessment/ | 30275 |
| 11 | (patient-centred outcome* or outcome assessment* or needs assessment* or symptom assessment* or health assessment* or assessment tool* or the person centred climate questionnaire).mp. [mp=title, abstract, original title, name of substance word, subject heading word, floating sub-heading word, keyword heading word, organism supplementary concept word, protocol supplementary concept word, rare disease supplementary concept word, unique identifier, synonyms] | 156824 |
| 12 | exp Decision Making/ | 201865 |
| 13 | exp Communication/ | 308239 |
| 14 | exp Patient-Centered Care/ | 20199 |
| 15 | (decision-making or patient-centred care or collaboration).mp. [mp=title, abstract, original title, name of substance word, subject heading word, floating sub-heading word, keyword heading word, organism supplementary concept word, protocol supplementary concept word, rare disease supplementary concept word, unique identifier, synonyms] | 286467 |
| 16 | exp quality of life/ | 194683 |
| 17 | (quality of life or QoL or life satisfaction).mp. [mp=title, abstract, original title, name of substance word, subject heading word, floating sub-heading word, keyword heading word, organism supplementary concept word, protocol supplementary concept word, rare disease supplementary concept word, unique identifier, synonyms] | 351607 |
| 18 | 1 or 2 or 3 or 4 or 5 or 6 or 7 | 393719 |
| 19 | 8 or 9 or 10 or 11 | 179520 |
| 20 | 12 or 13 or 14 or 15 or 16 or 17 | 1010853 |
| 21 | 18 and 19 and 20 | 2932 |

Note: this table includes the search terms for Medline database

Database(s): **APA PsycInfo**1806 to July Week 2 2020
Search Strategy:

| **#** | **Searches** | **Results** |
| --- | --- | --- |
| 1 | exp Dementia/ | 77248 |
| 2 | exp Alzheimer Disease/ | 46746 |
| 3 | exp cognitive impairment/ | 37068 |
| 4 | exp nursing home/ | 8628 |
| 5 | exp assisted living/ | 719 |
| 6 | (dementia or alzheimer* or cogniti* impairment or care home or nursing home or assisted living facilit*).mp. [mp=title, abstract, heading word, table of contents, key concepts, original title, tests & measures, mesh] | 143159 |
| 7 | exp Patient reported outcome measure/ | 306 |
| 8 | exp "treatment process and outcome measures"/ | 353 |
| 9 | exp Treatment outcomes/ | 122853 |
| 10 | exp Needs assessment/ | 4254 |
| 11 | (Person-centred care or person-centred outcomes or outcome assessment* or needs assessment* or symptom assessment* or health assessment* or assessment tool* or person centred climate questionnaire).mp. [mp=title, abstract, heading word, table of contents, key concepts, original title, tests & measures, mesh] | 41966 |
| 12 | exp Decision Making/ | 120462 |
| 13 | exp Communication/ | 304182 |
| 14 | exp Collaboration/ | 10764 |
| 15 | (decision-making or communication or collaboration or person-centred care).mp. [mp=title, abstract, heading word, table of contents, key concepts, original title, tests & measures, mesh] | 425261 |
| 16 | exp quality of life/ | 43074 |
| 17 | exp activities of daily living/ | 5977 |
| 18 | (quality of life or QoL or life satisfaction or activities of daily living).mp. [mp=title, abstract, heading word, table of contents, key concepts, original title, tests & measures, mesh] | 123325 |
| 19 | 1 or 2 or 3 or 4 or 5 or 6 | 145305 |
| 20 | 7 or 8 or 9 or 10 or 11 | 161512 |
| 21 | 12 or 13 or 14 or 15 or 16 or 17 or 18 | 719452 |
| 22 | 19 and 20 and 21 | 2502 |

Note: this table includes the search terms for Psycinfo database

**ASSIA database – search on 23/07/2020 – result = 2173**

((mainsubject.Exact("decision-making") OR mainsubject(communication) OR mainsubject("patient-centred care") OR ti,ab ("decision-making" OR communication OR "person-centred care" OR collaboration)) OR (mainsubject("quality of life") OR mainsubject("Activities of daily living") OR ti,ab("quality of life") OR ti,ab(QOL) OR ti,ab("life satisfaction") OR ti,ab("activities of daily living"))) AND (ti,ab("Person-centred outcomes" OR "outcome assessment" OR "needs assessment" OR "symptom assessment" OR "health assessment" OR "assessment tool" OR "person-centred assessment" OR "client-centre care questionnaire" OR "person-centred care assessment tool" OR "the person centred climate questionnaire") OR (mainsubject("decision-making") OR mainsubject(communication) OR mainsubject("patient-centred care") OR "decision-making" OR communication OR "patient-centred care")) AND (mainsubject(dementia) OR mainsubject(cognitive impairment) OR mainsubject("alzheimer's disease") OR mainsubject("nursing homes") OR mainsubject("care homes") OR ti,ab(dementia) OR ti,ab(cognitive impairment) OR ti,ab(alzheimer) OR ti,ab(care home) OR ti,ab(nursing home) OR ti,ab(assisted living facility))

CINAHL database:

| **#** | **Query** | **Results** |
| --- | --- | --- |
| S20 | S17 AND S18 AND S19 | 1,152 |
| S19 | S10 OR S11 OR S12 OR S13 OR S14 OR S15 OR S16 | 721,216 |
| S18 | S6 OR S7 OR S8 OR S9 | 78,251 |
| S17 | S1 OR S2 OR S3 OR S4 OR S5 | 149,336 |
| S16 | AB "Quality of life" OR AB QoL OR AB "health-related quality of life" OR AB "life satisfaction" OR AB "activities of daily living" | 122,238 |
| S15 | (MH "activities of daily living+) | 70,533 |
| S14 | (MH "quality of life+") | 118,855 |
| S13 | AB "decision-making" OR AB communication or AB "person-centred care" or AB collaboration | 169,772 |
| S12 | (MH "Communication+") | 275,485 |
| S11 | (MM "collaboration") | 15,460 |
| S10 | (MH "Decision Making+") | 125,194 |
| S9 | AB “Person-centred outcomes” OR AB “outcome assessment” OR AB “needs assessment” OR AB “symptom assessment” OR AB “health assessment” OR AB “assessment tool” OR AB "person-centred care assessment tool" OR AB "client-centre care questionnaire" | 17,626 |
| S8 | (MH "Patient-Reported Outcomes+") | 2,479 |
| S7 | (MH "Outcome Assessment+") | 43,614 |
| S6 | (MH "Needs Assessment+") | 17,581 |
| S5 | AB dementia OR AB alzheimer* OR AB "cogniti* impairment" OR AB "care home*" OR AB "nursing home*" OR "aged care facilit*" | 78,973 |
| S4 | (MH "Nursing Homes+") | 27,272 |
| S3 | (MH "Alzheimer's Disease+") | 32,121 |
| S2 | (MH "Cognition Disorders+") | 31,532 |
| S1 | (MH dementia+) | 72,770 |

Note: this table includes the search terms for CINAHL database

## Table S4: Data synthesis of how the use of PCOMs enables shared decision-making

| **Themes** | **Code** | **Components of shared decision-making** | **Evidence from papers** | | **Questions asked of data** |
| --- | --- | --- | --- | --- | --- |
| 1. **Knowing the person** | **The coming together of the care network** – *This involves the coming together of people involved in the care network of the person with dementia to facilitate a comprehensive, person centred assessment, often in a structured assessment to understand the person* | **Constructive network engagement -** It includes engaging with all relevant care network members to involve them in decision-making. The sub-elements are defining the participants, involving participants, and handling the nature of the interactions between participants. | The PAT questionnaire is filled out biyearly and on an as needed basis on all patients at two of the quarterly family conference meetings. The family conference meeting involves the interdisciplinary staff which consists of a family member (attends 60–70% of time), a nurse that has contact with the patient on the division, the resident care director (charge nurse that supervises all staff on the division), and social worker. **(Dahl 2008)**  Collaborative assessment between family and care home staff and between all care home staff. Care home staff identified that sometimes they had gaps in their knowledge of residents, particularly regarding to residents’ earlier lives. Findings from both family and professional participants suggested that using IPOS-Dem could address this through facilitating consultation with family in the assessment of residents  *‘Erm it sort of put you in the mind of, although we’re doing care plans and we’re doing report but it gives you a picture as well you know that you, you’re seeing a picture of a person when you’re doing this so yeah it do helps (Care home staff A3003)* **(Ellis-Smith 2018)**  Family members were involved throughout the assessment process by providing information related to premorbid patterns of function and interest in IADL and recreational activities. This input was critical in the development of realistic and relevant goals.  The information gathered from observations (structured using the APM), standardized assessment tools and family/ resident input was reviewed by the OT and TRS and used to develop one or two resident-specific goals **(Hartman 1997)**  ***PAINAD was [a] quick tool to use if family was there. (FG 2 n = 5) (Fry 2017)***  Provided a way for family members and the person with dementia to contribute to the assessment process by completing the measure/ or contributing to it.  The clinical services reported that, where possible, information was sought from the person themselves, with use of proxies for people lacking mental capacity to self-report. Of note, skin breakdown was the only symptom with no missing data. **(Kinley 2019)**  Using IdA, the nurses were asked to describe the specific situations in which the resident screamed. When they realized that this occurred only when the resident was provided with incontinence materials in the bathroom, the nursing staff began asking relatives for possible explanations for this behavior.  Nursing staff also reported that they avoided talking with family members to avoid conflicts with residents’ relatives. However, when they did talk with the family members, the staff received positive feedback from relatives for ‘‘showing more interest’ in the resident. **(Holle 2014)**  Care professionals discuss CAPs and Scales in team/with the patient/with the family **(Hermans 2014)**  ADMIT Me has the potential to prevent frustration during hospitalization, promote holistic care, and enable nurses to have quality conversation with the patient's family and health care team regarding the patient's health conditions **(Moore 2017)**  The person with dementia (if possible) and/or their caregiver identified their readiness to change behaviour for an identified high falls risk factor using an adapted version of the Trans-Theoretical Model for Behaviour Change (McNulty, Johnson, Poole, & Winkle, 2003; **(Meyer 2019)**  Intervention patients and companions endorsed an active communication role for companions in helping the patient understand what the clinician says or means (90%). Reminding the patient to ask questions (84%), asking questions directly to the clinician (84%), or listening and taking notes (82%). The majority (84%) endorsed three or more of these behaviors. **(Wolff 2018)** | | **At what point did care network come together?**  **At the point of assessment – to support the assessment process through active participation**  *The PAT questionnaire is filled out biyearly and on an as needed basis on all patients at two of the quarterly family conference meetings. (Dahl 2008)*  *Collaborative assessment between family and care home staff and between all care home staff. Care home staff identified that sometimes they had gaps in their knowledge of residents, particularly regarding to residents’ earlier lives. (Ellis-Smith 2018)*  *Family members were involved throughout the assessment process by providing information related to premorbid patterns of function and interest in IADL and recreational activities. (Hartman 1997)*  *Provided a way for family members and the person with dementia to contribute to the assessment process by completing the measure/ or contributing to it. (Kinley 2019)*  *Patients and companions each identified 4.4 health issues (range 0–12) as priorities for the visit agenda on average (Wolff 2018)*  *Patients and companions each identified 4.4 health issues (range 0–12) as priorities for the visit agenda on average (Meyer 2019)*  **Was the assessment process structured? E.g., how frequently? Were informal carers always involved?**  **Mainly structured with suggested frequency and how it should be completed**  *The PAT questionnaire is filled out biyearly and on an as needed basis on all patients at two of the quarterly family conference meetings. (Dahl)*  *Over the course of the year, these care professionals filled out the interRAI PC every three months for all residents identified as eligible. (Hermans 2018)*  *The instruction manual recommends that IPOS-Dem is used monthly at the time of care plans, or flexibly at times of resident change. (Ellis-Smith 2018)*  *The information gathered from observations (structured using the APM), standardized assessment tools and family/ resident input was reviewed by the OT and TRS and used to develop one or two resident-specific goals. (Hartman 1997)*  *During the 6-month intervention period, support was provided through regular home visits (up to once per month for 6 months as required), and phone call and/or email contact for questions/clarification between visits. After the first 6-month phase an action plan for the second 6 months was developed by the KB together with the person with dementia and their caregiver. This detailed plan built on the strategies undertaken in the first 6 months. (Meyer 2019)*  *The checklist involves two activities. The first activity involves clarifying expectations for the companion’s role, from a list of behaviors previously found to be beneficial during medical visits. The second activity asks patient and companion to each identify concerns about the patient’s health and decide together which to discuss with the clinician. (Wolff 2018)*  **Who were the carers?**  **unknown**  **How often was the person with dementia involved in the process and how severe was the dementia?**  **Involvement evident in a few papers. In cases where people with dementia are involved, they were in the mild-moderate stage of dementia**  *The information gathered from observations (structured using the APM), standardized assessment tools and family/ resident input was reviewed by the OT and TRS and used to develop one or two resident-specific goals. (Hartman 1997)*  *The checklist involves two activities. The first activity involves clarifying expectations for the companion’s role, from a list of behaviors previously found to be beneficial during medical visits. The second activity asks patient and companion to each identify concerns about the patient’s health and decide together which to discuss with the clinician. (Wolff 2018)*  *Facilitated awareness of problems by care staff and family members not previously identified people with dementia enjoyed the opportunity to discuss problems/concerns/care/treatment (Kinley 2019)*  *The person with dementia (if possible) and/or their caregiver identified their readiness to change behaviour for an identified high falls risk factor (Meyer 2019)* |
| 1. **Identifying problems, priorities for care and treatment, and goal setting** | **Highlighting problems, discussion of treatment and care, and goal setting, –** This stage involved having a discussion around what the needs are, particularly what are the priorities for the person with dementia and their family, and setting goals. | **Recognising the need for a decision now -** It includes negotiating with the network about emerging decision topics, arising from the differences between participants in terms of recognizing problems. | Family/carers sometimes alerted the nurses of the need for analgesia and of changes in the person’s pain levels. **(Fry 2017)**  People with dementia enjoyed the opportunity to discuss problems/concerns/care/treatment (**Kinley 2019)**  The most important risk factor for the person with dementia and/or their caregiver was addressed first, offering strategies. **(Meyer 2019)**  Patients and companions each identified 4.4 health issues (range 0–12) as priorities for the visit agenda on average (Fig. 3). Patient–companion visit priorities were discordant for 4 of the 12 health issues. Patients were more likely than companions to prioritize memory (59.2 versus 38.8%; p = 0.012) and stress, worry, or feeling sad or blue (42.9 versus 24.5%; p = 0.013). Companions were more likely than patients to prioritize safety at home or when driving (36.7 versus 18.4%; p = 0.039) and changes in personality or behavior (32.7 versus 16.3%; p = 0.011). **(Wolff 2018)** | | **Who was involved? The care network, the PwD, the informal carers and care professionals**  *Family/carers sometimes alerted the nurses of the need for analgesia and of changes in the person’s pain levels (Fry 2017)*  *People with dementia enjoyed the opportunity to discuss problems/concerns/care/treatment (Kinley 2019)*  *Patients and companions each identified 4.4 health issues (range 0–12) as priorities for the visit agenda on average (Fig. 3). Patient–companion visit priorities were discordant for 4 of the 12 health issues. (Wolff 2018)*  *The most important risk factor for the person with dementia and/or their caregiver was addressed first, offering strategies. (Meyer 2019)*  **What is the intention of this stage?**  **To raise awareness in changes to condition, identify and discuss care and treatment** |
|  |  | **Defining what needs to be decided on –**  It includes explicating decision topics and the related goals, and choosing which problem needs to be addressed now | Perhaps the most significant impact of GAS occurred in the focus of treatment planning. Rather than planning global activity programs, emphasis was shifted to a thorough assessment of each individual's skills as well as family/resident involvement in identifying personal interests and goals. **(Hartman 1997)**  The most important risk factor for the person with dementia and/or their caregiver was addressed first, offering strategies. Action plans and goals were set according to their choice of a variety of strategies presented by the KB to address the prioritised risk factor/s **(Meyer 2019)**  Patients and companions each identified 4.4 health issues (range 0–12) as priorities for the visit agenda on average (Fig. 3). Patient–companion visit priorities were discordant for 4 of the 12 health issues. Patients were more likely than companions to prioritize memory (59.2 versus 38.8%; p = 0.012) and stress, worry, or feeling sad or blue (42.9 versus 24.5%; p = 0.013). Companions were more likely than patients to prioritize safety at home or when driving (36.7 versus 18.4%; p = 0.039) and changes in personality or behavior (32.7 versus 16.3%; p = 0.011). **(Wolff 2018)** | | **What types of goals were set?**  **Specific and personalized**  *Rather than planning global activity programs, emphasis was shifted to a thorough assessment of each individual's skills as well as family/resident involvement in identifying personal interests and goals. (Hartman 1997)*  *Action plans and goals were set according to their choice of a variety of strategies presented by the KB to address the prioritised risk factor/s (Meyer 2019)* |
|  |  | **Developing alternatives -** Includes developing alternatives together with the network, often by  trial and error. Care networks usually perceive a lack of readily available  alternatives. Alternatives therefore depend on the possibilities and  knowledge of the care network. | Discrepancy between the person with dementia and their caregiver was resolved via further discussion of pros and cons of options. Following discussion of high falls risk factors as outlined above, strategy selection occurred through three main channels: (1) referrals (e.g. physiotherapy assessment, assistive devices through occupational therapy and optometry review); (2) information and contacts provided for resources (e.g. self-review of home hazards, continence aids and dietary guidelines for older adults); and (3) other advice/education (e.g. non-pharmacological approaches for managing behaviours of concern, toileting routine and consistent routines/meal time practices) (see detail in Supplementary Table online). Identification of potential strategies occurred via the following flexible approach (Figure 2):  • Discussion of pros and cons of various strategies;  • Discussion of decision-making options (accounting for individual needs and preferences);  • Provision of advice/education (as deemed clinically relevant by the KB);  • Provision of written and/or DVD resources (with reflection on the content if/when the dyad wished to discuss);  **(Meyer 2019)** | | **Who was involved?**  *Discrepancy between the person with dementia and their caregiver was resolved via further discussion of pros and cons of options (Meyer 2019)*  **How were alternatives developed?**  **Through discussions (not trying out)**  *Identification of potential strategies occurred via the following flexible approach (Figure 2):*  *• Discussion of pros and cons of various strategies;*  *• Discussion of decision-making options (accounting for individual needs and preferences);*  *• Provision of advice/education (as deemed clinically relevant by the KB);*  *• Provision of written and/or DVD resources (with reflection on the content if/when the dyad wished to discuss) (Meyer 2019)* |
|  |  | **Constructing preferences though deliberation and trying alternatives** | Discrepancy between the person with dementia and their caregiver was resolved via further discussion of pros and cons of options. Following discussion of high falls risk factors as outlined above, strategy selection occurred through three main channels: (1) referrals (e.g. physiotherapy assessment, assistive devices through occupational therapy and optometry review); (2) information and contacts provided for resources (e.g. self-review of home hazards, continence aids and dietary guidelines for older adults); and (3) other advice/education (e.g. non-pharmacological approaches for managing behaviours of concern, toileting routine and consistent routines/meal time practices) (see detail in Supplementary Table online). Identification of potential strategies occurred via the following flexible approach (Figure 2):  • Discussion of pros and cons of various strategies;  • Discussion of decision-making options (accounting for individual needs and preferences);  • Provision of advice/education (as deemed clinically relevant by the KB);  • Provision of written and/or DVD resources (with reflection on the content if/when the dyad wished to discuss);  **(Meyer 2019)** | | **How were preferences identified?**  **Through discussion**  *Identification of potential strategies occurred via the following flexible approach (Figure 2):*  *• Discussion of pros and cons of various strategies;*  *• Discussion of decision-making options (accounting for individual needs and preferences);*  *• Provision of advice/education (as deemed clinically relevant by the KB);*  *• Provision of written and/or DVD resources (with reflection on the content if/when the dyad wished to discuss) (Meyer 2019)* |
| 1. **Re-evaluating decisions** |  | **Evaluating decision-making** | Most importantly we had PAT questionnaires completed and discussed on all residents in the facility over the year evaluated. This means that each individual was discussed with family members and staff. Medications were evaluated. Possible side effects to medications were discussed and changes in medication were made if necessary and all of this was documented on a permanent record for the chart. **(Dahl 2008)**  Based on the CAPS results from the interRAI PC assessment and the accompanying manuals, care professionals were able to evaluate, adapt, and design individual care plans. **(Hermans 2018)** | **How often did evaluation happen?**  **What happened as a result?**  **Care and treatment plan adapted if necessary**  *Most importantly we had PAT questionnaires completed and discussed on all residents in the facility over the year evaluated. This means that each individual was discussed with family members and staff. Medications were evaluated. (Dahl 2008)*  *Based on the CAPS results from the interRAI PC assessment and the accompanying manuals, care professionals were able to evaluate, adapt, and design individual care plans. (Hermans 2018)* | |

Note: This table details the data analysis of how PCOM use leads to shared decision-making and impacts outcomes of care.

## Table S5 – Implementation processes

| **INTERVENTION CHARACTERISTICS** | **Example quotes** |
| --- | --- |
| ***Ease of use*** | Care home staff participants overwhelmingly reported that IPOS-Dem was easy to use and did not feel that training was required. **(Ellis-Smith 2017)** |
| ***Expertise of those using the intervention*** | Consequently, pain assessment for this patient group often relied on the nurse’s experience in judging the need for pain management. **(Fry 2017)**  In many nursing homes, participants in those case conferences were not well educated in nursing. They reported a lack of specific knowledge regarding dementia, which made it difficult to establish a link between the issues in IdA and the challenging behavior of residents with dementia.  ***There is a lack of specific knowledge about dementia in this ward . . . () we do not have that any more. Our daily routine is very much simpler. (group interview, team D1, interviewee 1)***  **(Holle 2014)**  Within this, a knowledge broker (KB) role is of benefit to make sense of the evidence and facilitate effective partnerships (Armstrong et al.,2011). To be effective (in the context of falls prevention, or other health-related issues), a KB should be based within an existing health/aged care service, strategically positioned to understand the barriers and opportunities inherent in the delivery and receipt of falls prevention knowledge relevant to their clients, in the local environmental context (Meyer, Dow, Hill, Tinney,& Hill, 2016). **(Meyer 2019)** |
| ***Availability of a manual*** | During the implementation period, participating care homes were given IPOS-Dem and asked to use it, according to the instruction manual with recruited residents. The instruction manual recommends that IPOS-Dem is used monthly at the time of care plans, or flexibly at times of resident change. **(Ellis-Smith 2018)**  When asked about dissemination of the tool, one first responder suggested using yellow paper for the tool and instructing used to hand it on the refrigerator. Although it would stand out, it was determined that it may be impractical to send the tool via email and ask caregivers to print on yellow paper. However, the tool states that it should be posted on the refrigerator. **(Moore 2017)** |
| ***Structure to the process*** | One explanation given for the lack of concentration was the difficulty in moderating a case conference for someone who was personally involved in the care of the person being discussed. Small groups of 2 to 3 people or case conferences in teams with a moderator who was specifically identified in all meetings were easier to manage. Participants also appreciated case conferences that were moderated by 1 member of the research team. **(Holle 2014)** |
| ***Availability of technology*** | Touchscreen technology, while not essential, was identified as a potential key facilitator in completing IPOS-Dem, storing records, monitoring over time and communication including online access for family members. This technology is becoming increasingly common in using measures in routine care [17] and may support implementation particularly if it facilitates measure completion, storing, retrieving and analysis of scores [68]. **(Ellis-Smith 2018)** |
| **STAFF NEEDS AND EXPERTISE** |  |
| ***Attitude towards the intervention*** | Leadership was seen as required to support adoption by all care home staff, ensuring that care home staff remember to use the measure, and ensuring they understand its purpose; thus ensuring that the measure is recognised as a valued tool to support care provision despite additional time burden **(Ellis-Smith 2018)**  Another caregiver responded, ***"something like this I think would be immensely helpful. If something like this existed, it would make stepping into the role as a caregiver so much easier."*** A third participant added, ***"ADMIT Me allows all health care professionals to be up-to-date with the patient."***  ***"This would be huge for someone with no one"*** (e.g. family support). Another nurse added, ***"The medical and behavioural pieces are the most helpful on the form."*** One nurse was particularly enthusiastic. When asked about dissemination of the tool, she stated, ***"This should be in every physician's office, it should be handed out at discharge."***  When asked, ***"what is the biggest barrier to care transition?",*** the first responders discussed not knowing the patient. One first responder commented:  The nurse wasn't familiar with the patient at all. The med reconciliation wasn't right. Baseline [of the patient] was very combative. She [the patient] was screaming the entire time and [we] had no idea how to calm her down. If we had this, like where it says behaviours and what triggers them, we could have avoided this from happening. **(Moore 2017)** |
| ***Integration with other services*** | Finally, staff may benefit from integration with primary and community health care services to support facilitation and shared organisational processes in care delivery. This type of integrated approach between care homes and health care services could facilitate use of IPOS-Dem into routine care and organisational processes, how to respond to symptoms and concerns, and support management and treatment of symptoms and concerns [16, 66]. **(Ellis-Smith 2018)** |
| ***Leadership and organisational support*** | Managers and care home staff considered leadership as essential in implementing IPOS-Dem to facilitate integration into routine care processes through e.g. supervision, care planning. Leadership was seen as required to support adoption by all care home staff, ensuring that care home staff remember to use the measure, and ensuring they understand its purpose; thus ensuring that the measure is recognised as a valued tool to support care provision despite additional time burden **(Ellis-Smith 2018)**  We found that leadership engagement at all phases was essential in implementing IPOS-Dem, corroborating existing evidence [68, 69] **(Ellis-Smith 2018)**  Interventions that were adopted by the nursing staff in relation to the challenging behavior of residents with dementia in the dementia-specific case conferences with IdA were rejected by staff working in administration or domestic management or at the reception desk of the nursing home. These staff members pointed to the breach of duty of care and the legal responsibility to care for the resident. Nursing staff could not withstand the pressure from the other professionals and reversed their decisions. **(Holle 2014)** |
| ***Resources*** |  |
| Staffing | Due to the lack of staffing, case conferences were often held in the rooms of the nursing service, so a spatial separation from the working area was not available. Participants also reported that management promised them additional staff during the dementia-specific case conferences with IdA, but this promise was not realized during the course of the study. **(Holle 2014)** |
| Busyness, heavy workload and time constraints | Leadership was seen as required to support adoption by all care home staff, ensuring that care home staff remember to use the measure, and ensuring they understand its purpose; thus ensuring that the measure is recognised as a valued tool to support care provision despite additional time burden **(Ellis-Smith 2018)**  To some extent, pain cues were ignored and pain assessment and management were neglected in the presence of significant communication barriers because of the additional time required to address this issue. **(Fry 2017)**  Research shows that filling out the interRAI instruments is an extensive, laborious, and time-consuming process. Care professionals do not always have sufficient time to complete these instruments, especially not in a nursing home, where there is such a heavy workload (Hermans et al., 2016b; Devriendt et al., 2013; Vanneste & Declercq, 2014). After this period, the perceived and actual waste of time may be reduced. **(Hermans 2018)**  It took about a year to implement the interRAI PC and the BelRAI web application in the nursing homes (Hermans et al., 2016b), and some nursing homes did not have sufficient time to discuss and work with the interRAI PC results (CAPs and scales) beyond merely registering. Hence, they did not use the results to develop, evaluate, and adjust care plans. **(Hermans 2018)**  Another factor that affected the implementation of the dementia-specific case conferences with IdA was the available time resources of nursing teams. Nurses had significant time pressure due to high workloads. Therefore, they could not attend monthly case conferences with durations of 90 to 120 minutes. In addition to a lack of time, nurses also identified limitations in the application of IdA within the dementia-specific case conference. They rated IdA as too long in relation to the time available for case conferences, particularly because additional time had to be considered for the receipt of information from relatives or doctors following those case conferences. Due to lack of time and resources, no preparation or follow-up was possible. **(Holle 2014)**  Nurses spoke of the challenge, in the busy ED environment, to ensure older people with CI were assessed and reassessed for ongoing pain.  ***As nurses we do get caught up in the routine. Patients arrive, we undress them, we do the ECG and we do full set [of vital signs] and right documentation. And sometimes I have to say to a couple of new people that this person is in pain and why don’t we get them some analgesia first, then x-ray or ECG later. They do not have chest pain. They have broken legs so let’s give analgesia first. (FG 5 n = 6)* (Fry 2017)** |
| **Training requirement (Ellis-Smith, Higginson et al. 2018; Kinley, Ellis-Smith et al. 2019)** | Nonetheless, managers reported the potential benefit of staff training on how IPOS Dem may support care. Provision of training on how IPOS-Dem may be integrated into care processes may also supports its implementation and use in routine care. **(Ellis-Smith 2018)**  Some nurses were able to answer the IdA questions but could not link the information with residents’ challenging behavior.In the dementia-specific case conferences with IdA that were accompanied by the research team, the relationship between the IdA questions and the residents’ challenging behavior often had to be explained by 1 person on the research team. **(Holle 2014)**  The process commenced with ‘face-to-face’ training by authors CES and JK, with the clinical service leader(s) on the outcome measures to be implemented, using, for example, case scenarios to explain use of the measures in routine care. The clinical leads then trained the staff within their respective organisations to implement the measures using team meetings and role modelling, and tailoring implementation to the respective clinical setting and population. JK/CES provided advice and support throughout the audit process. **(Kinley 2019)** |
| **INTERVENTION CHARACTERISTICS** | **Example quotes** |
| ***Ease of use*** | Care home staff participants overwhelmingly reported that IPOS-Dem was easy to use and did not feel that training was required. **(Ellis-Smith 2017)** |
| ***Expertise of those using the intervention*** | Consequently, pain assessment for this patient group often relied on the nurse’s experience in judging the need for pain management. **(Fry 2017)**  In many nursing homes, participants in those case conferences were not well educated in nursing. They reported a lack of specific knowledge regarding dementia, which made it difficult to establish a link between the issues in IdA and the challenging behavior of residents with dementia.  ***There is a lack of specific knowledge about dementia in this ward . . . () we do not have that any more. Our daily routine is very much simpler. (group interview, team D1, interviewee 1)***  **(Holle 2014)**  Within this, a knowledge broker (KB) role is of benefit to make sense of the evidence and facilitate effective partnerships (Armstrong et al.,2011). To be effective (in the context of falls prevention, or other health-related issues), a KB should be based within an existing health/aged care service, strategically positioned to understand the barriers and opportunities inherent in the delivery and receipt of falls prevention knowledge relevant to their clients, in the local environmental context (Meyer, Dow, Hill, Tinney,& Hill, 2016). **(Meyer 2019)** |
| ***Availability of a manual*** | During the implementation period, participating care homes were given IPOS-Dem and asked to use it, according to the instruction manual with recruited residents. The instruction manual recommends that IPOS-Dem is used monthly at the time of care plans, or flexibly at times of resident change. **(Ellis-Smith 2018)**  When asked about dissemination of the tool, one first responder suggested using yellow paper for the tool and instructing used to hand it on the refrigerator. Although it would stand out, it was determined that it may be impractical to send the tool via email and ask caregivers to print on yellow paper. However, the tool states that it should be posted on the refrigerator. **(Moore 2017)** |
| ***Structure to the process*** | One explanation given for the lack of concentration was the difficulty in moderating a case conference for someone who was personally involved in the care of the person being discussed. Small groups of 2 to 3 people or case conferences in teams with a moderator who was specifically identified in all meetings were easier to manage. Participants also appreciated case conferences that were moderated by 1 member of the research team. **(Holle 2014)** |
| ***Availability of technology*** | Touchscreen technology, while not essential, was identified as a potential key facilitator in completing IPOS-Dem, storing records, monitoring over time and communication including online access for family members. This technology is becoming increasingly common in using measures in routine care [17] and may support implementation particularly if it facilitates measure completion, storing, retrieving and analysis of scores [68]. **(Ellis-Smith 2018)** |
| **STAFF NEEDS AND EXPERTISE** |  |
| ***Attitude towards the intervention*** | Leadership was seen as required to support adoption by all care home staff, ensuring that care home staff remember to use the measure, and ensuring they understand its purpose; thus ensuring that the measure is recognised as a valued tool to support care provision despite additional time burden **(Ellis-Smith 2018)**  Another caregiver responded, ***"something like this I think would be immensely helpful. If something like this existed, it would make stepping into the role as a caregiver so much easier."*** A third participant added, ***"ADMIT Me allows all health care professionals to be up-to-date with the patient."***  ***"This would be huge for someone with no one"*** (e.g. family support). Another nurse added, ***"The medical and behavioural pieces are the most helpful on the form."*** One nurse was particularly enthusiastic. When asked about dissemination of the tool, she stated, ***"This should be in every physician's office, it should be handed out at discharge."***  When asked, ***"what is the biggest barrier to care transition?",*** the first responders discussed not knowing the patient. One first responder commented:  The nurse wasn't familiar with the patient at all. The med reconciliation wasn't right. Baseline [of the patient] was very combative. She [the patient] was screaming the entire time and [we] had no idea how to calm her down. If we had this, like where it says behaviours and what triggers them, we could have avoided this from happening. **(Moore 2017)** |
| ***Integration with other services*** | Finally, staff may benefit from integration with primary and community health care services to support facilitation and shared organisational processes in care delivery. This type of integrated approach between care homes and health care services could facilitate use of IPOS-Dem into routine care and organisational processes, how to respond to symptoms and concerns, and support management and treatment of symptoms and concerns [16, 66]. **(Ellis-Smith 2018)** |
| ***Leadership and organisational support*** | Managers and care home staff considered leadership as essential in implementing IPOS-Dem to facilitate integration into routine care processes through e.g. supervision, care planning. Leadership was seen as required to support adoption by all care home staff, ensuring that care home staff remember to use the measure, and ensuring they understand its purpose; thus ensuring that the measure is recognised as a valued tool to support care provision despite additional time burden **(Ellis-Smith 2018)**  We found that leadership engagement at all phases was essential in implementing IPOS-Dem, corroborating existing evidence [68, 69] **(Ellis-Smith 2018)**  Interventions that were adopted by the nursing staff in relation to the challenging behavior of residents with dementia in the dementia-specific case conferences with IdA were rejected by staff working in administration or domestic management or at the reception desk of the nursing home. These staff members pointed to the breach of duty of care and the legal responsibility to care for the resident. Nursing staff could not withstand the pressure from the other professionals and reversed their decisions. **(Holle 2014)** |
| ***Resources*** |  |
| Staffing | Due to the lack of staffing, case conferences were often held in the rooms of the nursing service, so a spatial separation from the working area was not available. Participants also reported that management promised them additional staff during the dementia-specific case conferences with IdA, but this promise was not realized during the course of the study. **(Holle 2014)** |
| Busyness, heavy workload and time constraints | Leadership was seen as required to support adoption by all care home staff, ensuring that care home staff remember to use the measure, and ensuring they understand its purpose; thus ensuring that the measure is recognised as a valued tool to support care provision despite additional time burden **(Ellis-Smith 2018)**  To some extent, pain cues were ignored and pain assessment and management were neglected in the presence of significant communication barriers because of the additional time required to address this issue. **(Fry 2017)**  Research shows that filling out the interRAI instruments is an extensive, laborious, and time-consuming process. Care professionals do not always have sufficient time to complete these instruments, especially not in a nursing home, where there is such a heavy workload (Hermans et al., 2016b; Devriendt et al., 2013; Vanneste & Declercq, 2014). After this period, the perceived and actual waste of time may be reduced. **(Hermans 2018)**  It took about a year to implement the interRAI PC and the BelRAI web application in the nursing homes (Hermans et al., 2016b), and some nursing homes did not have sufficient time to discuss and work with the interRAI PC results (CAPs and scales) beyond merely registering. Hence, they did not use the results to develop, evaluate, and adjust care plans. **(Hermans 2018)**  Another factor that affected the implementation of the dementia-specific case conferences with IdA was the available time resources of nursing teams. Nurses had significant time pressure due to high workloads. Therefore, they could not attend monthly case conferences with durations of 90 to 120 minutes. In addition to a lack of time, nurses also identified limitations in the application of IdA within the dementia-specific case conference. They rated IdA as too long in relation to the time available for case conferences, particularly because additional time had to be considered for the receipt of information from relatives or doctors following those case conferences. Due to lack of time and resources, no preparation or follow-up was possible. **(Holle 2014)**  Nurses spoke of the challenge, in the busy ED environment, to ensure older people with CI were assessed and reassessed for ongoing pain.  ***As nurses we do get caught up in the routine. Patients arrive, we undress them, we do the ECG and we do full set [of vital signs] and right documentation. And sometimes I have to say to a couple of new people that this person is in pain and why don’t we get them some analgesia first, then x-ray or ECG later. They do not have chest pain. They have broken legs so let’s give analgesia first. (FG 5 n = 6)* (Fry 2017)** |
| **Training requirement and continuous support** | Nonetheless, managers reported the potential benefit of staff training on how IPOS Dem may support care. Provision of training on how IPOS-Dem may be integrated into care processes may also supports its implementation and use in routine care. **(Ellis-Smith 2018)**  Some nurses were able to answer the IdA questions but could not link the information with residents’ challenging behavior.In the dementia-specific case conferences with IdA that were accompanied by the research team, the relationship between the IdA questions and the residents’ challenging behavior often had to be explained by 1 person on the research team. **(Holle 2014)**  The process commenced with ‘face-to-face’ training by authors CES and JK, with the clinical service leader(s) on the outcome measures to be implemented, using, for example, case scenarios to explain use of the measures in routine care. The clinical leads then trained the staff within their respective organisations to implement the measures using team meetings and role modelling, and tailoring implementation to the respective clinical setting and population. JK/CES provided advice and support throughout the audit process. **(Kinley 2019)**  Two feedback sessions to each of the clinical services were planned to improve the care they provided and evidence this. The initial session (at 3 months) intended to support the audit leads and answer questions regarding the audit process. The second session (at 6 months), with all staff involved in the audit, explored their experiences using the measures in routine practice. **(Kinley 2019)**  Finally, a summary of the results for each service, incorporating staff feedback, was shared with each clinical service and confirmation on accuracy/ completeness obtained. **(Kinley 2019)** |
| **FAMILY NEEDS** |  |
| ***Accessibility of intervention*** | Care home staff also identified the practical challenges of making IPOS-Dem accessible for family and external health professionals. **(Ellis-Smith 2018)** |

Note: This table details the implementation requirements for PCOM use in dementia care

| Table S6: Mixed Method Appraisal Tool | | | | | | | | | | | | | | | | | | | | | | | | | | | | | | |
| --- | --- | --- | --- | --- | --- | --- | --- | --- | --- | --- | --- | --- | --- | --- | --- | --- | --- | --- | --- | --- | --- | --- | --- | --- | --- | --- | --- | --- | --- | --- |
| **Author (year)** | **All studies** | | **Qualitative papers only  *'Yes', 'No', 'Can't tell' or add comment*** | | | | | | **Quantitative randomized controlled trials  *'Yes', 'No', 'Can't tell' or add comment*** | | | | | **Quantitative nonrandomized  *'Yes', 'No', 'Can't tell' or add comment*** | | | | | **Quantitative descriptive  *'Yes', 'No', 'Can't tell' or add comment*** | | | | | **Mixed methods  *'Yes', 'No', 'Can't tell' or add comment*** | | | | | |  |
|  | S1. Are there clear research questions? | S2. Do the collected data allow to address the research questions? | 1.1. Is the qualitative approach appropriate to answer the research question? | 1.2. Are the qualitative data collection methods adequate to address the research question? | | 1.3. Are the findings adequately derived from the data? | 1.4. Is the interpretation of results sufficiently substantiated by data? | 1.5. Is there coherence between qualitative data sources, collection, analysis and interpretation? | 2.1. Is randomization appropriately performed? | 2.2. Are the groups comparable at baseline? | 2.3. Are there complete outcome data? | 2.4. Are outcome assessors blinded to the intervention provided? | 2.5 Did the participants adhere to the assigned intervention? | 3.1. Are the participants representative of the target population? | 3.2. Are measurements appropriate regarding both the outcome and intervention (or exposure)? | 3.3. Are there complete outcome data? | 3.4. Are the confounders accounted for in the design and analysis? | 3.5. During the study period, is the intervention administered (or exposure occurred) as intended? | 4.1. Is the sampling strategy relevant to address the research question? | 4.2. Is the sample representative of the target population? | 4.3. Are the measurements appropriate? | 4.4. Is the risk of nonresponse bias low? | 4.5. Is the statistical analysis appropriate to answer the research question? | 5.1. Is there an adequate rationale for using a mixed methods design to address the research question? | 5.2. Are the different components of the study effectively integrated to answer the research question? | 5.3. Are the outputs of the integration of qualitative and quantitative components adequately interpreted? | 5.4. Are divergences and inconsistencies between quantitative and qualitative results adequately addressed? | 5.5. Do the different components of the study adhere to the quality criteria of each tradition of the methods involved? |  |  |
| Hartman (1997) | Yes | Yes |  |  |  | |  |  |  |  |  |  |  | Yes | Yes | Can't tell | Can't tell | Can't tell |  |  |  |  |  |  |  |  |  |  |  |  |
| Ellis-Smith (2018) | Yes | Yes | Yes | Yes | Yes | | Yes | Yes |  |  |  |  |  |  |  |  |  |  |  |  |  |  |  |  |  |  |  |  |  |  |
| Fry (2017) | Yes | Yes | Yes | Yes | Yes | | Yes | Yes |  |  |  |  |  |  |  |  |  |  |  |  |  |  |  |  |  |  |  |  |  |  |
| Holle (2014) | Yes | Yes | Yes | Yes | Yes | | Yes | Yes |  |  |  |  |  |  |  |  |  |  |  |  |  |  |  |  |  |  |  |  |  |  |
| Meyer (2019) | Yes | Yes |  |  |  | |  |  |  |  |  |  |  | Yes | Yes | Can't tell | Can’t tell | Can't tell |  |  |  |  |  |  |  |  |  |  |  |  |
| Hermans (2018) | Yes | Yes |  |  |  | |  |  |  |  |  |  |  | Yes | Yes | Yes | Can't tell | Yes |  |  |  |  |  |  |  |  |  |  |  |  |
| Moore (2017) | Yes | Yes | Yes | Yes | Can't tell | | Yes | Yes |  |  |  |  |  |  |  |  |  |  |  |  |  |  |  |  |  |  |  |  |  |  |
| Wolff (2018) | Yes | Yes |  |  |  | |  |  | Yes | Can't tell | Yes | Yes | Yes |  |  |  |  |  |  |  |  |  |  |  |  |  |  |  |  |  |

Note: This table details the quality of studies included in the systematic review, using the Mixed Method Appraisal Tool (MMAT)

Ellis-Smith, C., I. J. Higginson, B. A. Daveson, L. A. Henson, C. J. Evans and O. behalf of BuildCARE (2018). "How can a measure improve assessment and management of symptoms and concerns for people with dementia in care homes? A mixed-methods feasibility and process evaluation of IPOS-Dem." PloS one **13**(7).

Kinley, J., C. Ellis-Smith, M. Hurt, K. McIvor and C. J. Evans (2019). "A collaborative approach in dementia care to improve clinical effectiveness and priorities for research through audit." International Journal of Palliative Nursing **25**(12): 588-595.
